# An adenovirus-vectored RVF vaccine confers complete protection against lethal RVFV challenge in A129 mice

**DOI:** 10.3389/fmicb.2023.1114226

**Published:** 2023-02-28

**Authors:** Meng Hao, Ting Bian, Guangcheng Fu, Yi Chen, Ting Fang, Chuanyi Zhao, Shuling Liu, Changming Yu, Jianmin Li, Wei Chen

**Affiliations:** ^1^Vaccine and Antibody Engineer Laboratory, Beijing Institute of Biotechnology, Beijing, China; ^2^Frontier Biotechnology Laboratory, Zhejiang University-Hangzhou Global Scientific and Technological Innovation Center, Hangzhou, China

**Keywords:** RVFV vaccine, glycoprotein, adenovirus serotype 5 vector, single-dose immunization, sterilizing protection, A129 mice

## Abstract

**Instruction:** Rift valley fever virus (RVFV) is a mosquito-transmitted bunyavirus that causes severe disease in animals and humans. Nevertheless, there are no vaccines applied to prevent RVFV infection for human at present. Therefore, it is necessary to develop a safe and effective RVFV vaccine.

**Methods:** We generated Ad5-GnGcopt, a replication-deficient recombinant Ad5 vector (human adenovirus serotype 5) expressing codon-optimized RVFV glycoproteins Gn and Gc, and evaluated its immunogenicity and protective efficacy in mice.

**Results and Discussion:** Intramuscular immunization of Ad5-GnGcopt in mice induces strong and durable antibody production and robust cellular immune responses. Additionally, a single vaccination with Ad5-GnGcopt vaccination can completely protect interferon-α/β receptor-deficient A129 mice from lethal RVFV infection. Our work indicates that Ad5-GnGcopt might represent a potential vaccine candidate against RVFV. However, further research is needed, first to confirm its efficacy in a natural animal host, and ultimately escalate as a potential vaccine candidate for humans.

## Introduction

Rift valley fever virus (RVFV) is a mosquito-borne zoonotic infectious pathogen that is highly pathogenic and a serious threat to human and animal health (Weaver and Reisen, 2010; Brustolin et al., 2017; Kraemer et al., 2019). RVFV causes severe disease in ruminants, such as sheep, goats, and cattle, which gives rise to abortion storms and a high fatality rate in newborn lambs and calves (Davies and Martin, 2006). In endemic areas, disease prevalence is generally associated with the surge in mosquito populations after heavy rains. It has been reported that several Aedes and Culex mosquitoes can transmit RVFV (Moutailler et al., 2008; Turell et al., 2008). The virus is mainly endemic in Africa; however, in 2000, the first cases outside the African continent were reported in the Arabian Peninsula (Balkhy and Memish, 2003). In 2016, China reported the first imported case of RVFV infection (Liu et al., 2017). Under the influence of climate change and globalization, the virus spread tends to expand outward (Bron et al., 2021). In addition to animals, RVFV is also infectious to humans. Humans can be infected through contact with contaminated tissues or bodily fluids of RVFV-infected animals or when bitten by virus-carrying mosquitoes (Ikegami and Makino, 2011). Most patients infected with RVF display a self-limiting, acute febrile illness, although a minority of these cases there may progress to neurologic disorders, blindness, or lethal hemorrhagic fever (Madani et al., 2003; Ikegami and Makino, 2011). There is increasing evidence that RVFV infection in pregnant women may lead to complications (Baudin et al., 2016; McMillen et al., 2018; Oymans et al., 2020). Despite its significant impact on human health and the economy, no safe and efficacious prophylactic or therapeutic treatment options are currently available to human.

Rift valley fever virus belongs to the *Phlebovirus* genus in the *Phenuiviridae* family and is an enveloped virus containing three negative-stranded RNA segments: L, M, and S (Chapman et al., 2021). The L segment encodes the viral RNA-dependent RNA polymerase. The S segment encodes the nucleocapsid and a nonstructural protein named NSs. The nucleocapsid can protect viral RNA from degradation. The NSs inhibits the host innate immune responses and is a major factor in determining viral virulence (Ly and Ikegami, 2016). The M segment encodes a glycoprotein precursor, which is then splited by host proteases to generate two glycoproteins, named Gn and Gc. Gn plays an important role in attaching to host cells, whereas Gc is involved in the fusion of the viral and endosomal membranes. These two glycoproteins, associated with a heterodimer (Freiberg et al., 2008; Huiskonen et al., 2009; Sherman et al., 2009), play a major part in the early stages of viral infection and have been shown to carry virus-neutralizing epitopes (Won et al., 2007; Kading et al., 2014; Chapman et al., 2021), which are the main targets for the development of novel RVF vaccines. In addition, the M segment also encodes a small 14 kDa protein named NSm, which suppresses virus-induced apoptosis (Won et al., 2007), and a large 78 kDa protein named LGp, which is important for virus replication and dissemination in mosquitoes (Kading et al., 2014; Kreher et al., 2014; Weingartl et al., 2014).

Vaccination is one of the most cost-optimal and effective ways to prevent and control Rift Valley fever. Therefore, there are multiple kinds of development strategies applied to Rift Valley fever vaccine, including live attenuated, inactivated, DNA, viral vectored, subunit, viral replicons and virus-like particles vaccine (Kitandwe et al., 2022). Among these vaccines, one live attenuated vaccine MP-12, one viral vector vaccine ChAdOx1 and two inactivated vaccines NDBR-103 and TSI-GSD-200 have been evaluated in clinical trials for humans. The live attenuated vaccine MP-12 could induce high levels of humoral immune response in several animal models, such as lambs, cow, and rhesus monkeys, and induce long-lasting humoral immune response in a phase 2 clinical trial for humans (Pittman et al., 2016). Nonetheless, some serious adverse events induced by MP-12 have also been reported. The vaccine was elucidated to induce hepatocellular degeneration and necrosis in calves (Wilson et al., 2014) and teratogenic effects and abortions in sheep (Hunter et al., 2002). The formalin-inactivated vaccine NDBR-103 elicited a relatively high immune response, and 40 of 43 persons produced neutralizing antibodies of log 2 or more after a three-dose primary series immunization (Randall et al., 1962). But it also raises some safety concerns that the vaccine seed virus of NDBR-103 was produced in inoculum containing infectious mouse serum and primary monkey kidney cells. It was reported that the inactivated vaccine TSI-GSD-200 was relative safe and could induce long-lasting RVFV-specific T-cell responses, but the vaccine needed multiple immunizations to induce and maintain protective immune response (Harmon et al., 2020).The last vaccine in human clinical trials is the ChAdOx1-GnGc, a vaccine based on a replication-deficient chimpanzee adenovirus vector, which could induce RVFV-neutralizing antibodies in mice, sheep, goats, and cattle. In additional, a recombinant human adenovirus serotype 5 vector-based RVFV vaccine (HAdV5-GnGc) was used as a control group to compare with ChAdOx1-GnGc, and the data showed that the ability of ChAdOx1-GnGc to induce an immune response in mice is lower than that of HAdV5-GnGc. Nevertheless, the *in vivo* protective activity of ChAdOx1-GnGc and HAdV5-GnGc was not compared (Warimwe et al., 2013). And it raises some human safety concerns that the Vaccine-induced Immune Thrombotic Thrombocytopenia (VITT) was induced by the ChAdOx1-S COVID-19 vaccine, which is based on the ChAdOx1vector (Makris and Pavord, 2022).

It is worth mentioning that our research group have also developed a RVFV vaccine based on the replication-competent recombinant human adenovirus serotype 4 (HAdV-4), and this vaccine could induce NAb against RVFV and cellular immune responses in mice and also provide complete protection for A129 mice challenged by RVFV (Bian et al., 2022). Nevertheless, the limitation in the application of Ad4 vector vaccine is its potential pathogenicity. There are several studies suggesting that HAdV-4 can cause respiratory disease and even pneumonia in children (Gopalkrishna et al., 2016; Thounaojam et al., 2016; Wang et al., 2016; Heo et al., 2018). On the contrary, the successful clinical use of the vaccines against Ebola virus and SARS-CoV-2 based on the replication-incompetent recombinant human adenovirus serotype 5 (HAdV-5) developed by our research group demonstrated that HAdV-5 vector is very safe for humans (Wu et al., 2020; Zhu et al., 2020).

In this study, we chose a replication-incompetent human adenovirus type 5-based RVFV vaccine (Ad5-GnGcopt) based on our previous experience with Ad5-vectored Ebola vaccine and Ad5-vectored SARS-CoV-2 vaccine (Zhu et al., 2017; Wu et al., 2020). Ad5-GnGcopt encodes the codon-optimized genes for mammalian expression of RVFV MP-12 Gn and Gc proteins. We investigate the humoral and cellular immune responses elicited by the vaccine and evaluate the protective efficacy of the candidate vaccine against RVFV challenge in the interferon-α/β receptor-deficient A129 mice model.

## Materials and methods

### Cells, viruses, and animals

Vero E6 cells and HEK293 cells were cultured in Dulbecco’s modified Eagle’s medium (DMEM; Gibco, Grand Island, NY, United States) supplemented with 10% fetal bovine serum (FBS; Gibco, Grand Island, NY, United States), 100 U/mL penicillin, and 100 mg/mL streptomycin at 37°C with 5% CO_2_. The RVFV MP-12 strain (rMP-12) and RVFV MP-12 strain expressing eGFP (rMP-12-eGFP) were rescued through the reverse genetics approach (Ikegami et al., 2006). The recombinant human adenovirus serotype 5 vector was developed and maintained by the Beijing Institute of Biotechnology (Hunter et al., 2002; Wilson et al., 2014; Harmon et al., 2020).

All animal experiments were approved by the Animal Welfare and Ethics Committee of the Academy of Military Medical Sciences (permit number: IACUC-SWGCYJS-2021-006, date of approval: 11 March 2021). Four to six weeks female BALB/c mice were obtained from SPF (Beijing) Biotechnology Co., Ltd. (Beijing, China). The BALB/c mice and interferon-α/β receptor-deficient A129 mice were housed and bred in individually ventilated cages and provided water and food. The mice RVFV challenge experiments were conducted in animal biosafety level 2 (ABSL2) facilities at the Academy of Military Medical Sciences, Beijing.

### Construction of adenovirus vectored vaccine expressing RVFV glycoproteins Gn and Gc

Ad5-GnGcopt, a replication-incompetent Ad5-vectored vaccine, was constructed as previously described (Zhu et al., 2015; Wu et al., 2020). The RVFV MP-12 strain glycoproteins Gn and Gc (accession number DQ380208.1) were selected as immunogen. A Kozak sequence and tissue plasminogen activator (tPA) signal peptide were added to five-terminal of the codon-optimized Gn and Gc gene to enhance the expression level of the glycoproteins in host cells. The genes were cloned into the shuttle plasmid of the AdMax adenovirus system (Microbix Biosystems, Mississauga, Canada); then, the shuttle plasmid with the target gene was co-transfected into HEK293 cells with the backbone plasmid (pBHGloxΔE1, 3Cre) using TurboFect transfection reagent (Thermo Scientific, Waltham, MA, United States). The transfected cells were collected, when the typical Ad-related cytopathic effects were viewed. Successful construction of the Ad5-GnGcopt was verified based on the sequence of Gn and Gc gene and the protein size of Gn and Gc. The infectious units of the recombinant virus were titrated using an AdenoX™ Rapid Titer Kit (Clontech, Mountain View, CA, United States) according to the manufacturer’s instructions.

### Western blotting

To analyze the expression of Gn and Gc mediated by Ad5-GnGcopt, we infected Vero E6 cells in six-well cell culture dishes with Ad5-GnGcopt at a multiplicity of infection (MOI) of 10. The culture supernatant was discarded, and cells were washed twice with ice-cold phosphate-buffered saline at pH 7.4 (PBS) and lysed with 150 μl of RIPA Lysis and Extraction Buffer (Thermo Scientific, Waltham, MA, United States) 48 h after infection. Then, the cell lysate was centrifuged, and the supernatant was collected, mixed with 6× Protein Loading Buffer (TransGen Biotech, Beijing, China), and electrophoresed on a 12% Bis-Tris protein gel (GenScript, Nanjing, China). Protein was subsequently transferred to polyvinylidene fluoride (PVDF) membranes. After blocked by 5% skim milk at room temperature (RT) for 1 h, the PVDF membranes were incubated with anti-Gn monoclonal antibody (working concentration: 1 μg/mL) or anti-Gc rabbit polyclonal antibody (working dilution: 1:3,000) at RT for 1 h. After wash three times, the corresponding HRP-secondary antibodies, of which working dilution both were 1:10,000, were added and incubated at RT for 1 h. After wash three times, the chemiluminescent substrate (Merck Millipore, Burlington, MA, United States) was used to incubated with membranes and images were acquired with iBright imaging system (Thermo Scientific, Waltham, MA, United States). The β-actin was used as an internal reference and detected on the same membrane with anti-β-actin antibody (HRP; Abcam, Cambridge, United Kingdom).

### Mice vaccination and challenge experiments

Female BALB/c mice aged 4–6 weeks were inoculated intramuscularly with 10^6^ IFU (low dose, *n* = 8), 10^7^ IFU (middle dose, *n* = 8), or 10^8^ IFU (high dose, *n* = 26) of Ad5-GnGcopt or PBS as a placebo control (*n* = 26) at day 0. The mice sera were collected at different time points to titrate the Gn- and Gc-specific IgG titration and the RVFV neutralizing antibody (NAb) titration, respectively. At 1, 2, and 3 weeks after inoculation, six mice in the high dose group or placebo group were sacrificed for germinal center responses and splenic cellular immune response detections.

For RVFV challenge experiments, the A129 mouse was selected as the animal model, and the experiment was conducted as previously described with minor adjustments (Lorenzo et al., 2010; Gommet et al., 2011). A129 mice (*n* = 4) were immunized with 10^6^ or 10^8^ IFU of Ad5-GnGcopt or placebo *via* the intramuscular route. Four weeks after immunization, mice were inoculated with 2 × 10^4^ TCID_50_ of RVFV (rMP-12 strain) by the intraperitoneal route. Animals were monitored for body weight changes and survival for 14 days, and their livers and spleens were collected at necropsy for viral load detection, histopathology assay, and immunohistochemistry staining.

### ELISA

To determine the RVFV Gn- and Gc-specific IgG, IgG1, or IgG2a titer in mice, RVFV Gn or Gc proteins were diluted in carbonate–bicarbonate buffer and coated in the 96-well high-binding microplates (Corning, NY, United States) with 0.2 μg/well at 4°C overnight. The plates were then blocked with PBS containing 2% BSA at 37°C for 1 h. After three rinses with PBST (PBS containing 0.2% Tween-20), serially diluted sera of mice in dilution buffer (PBST containing 0.2% BSA) were incubated with RVFV Gn or Gc proteins coated in the microplates at 37°C for 1 h. After that, the microplates were rinsed three times with PBST and incubated with the secondary antibodies at 37°C for 1 h, which target mouse IgG, IgG1, or IgG2a (Abcam, Cambridge, United Kingdom). After microplates rinsed three times, the TMB substrate solution (Solarbio, Beijing, China) was added in the microplates for 6 min at room temperature and the reaction was terminated by stop solution (Solarbio, Beijing, China). The optical density (OD) was measured at 450/630 nm (OD450/OD630; SPECTRA, Molecular Device, San Jose, CA, United States). We defined the endpoint titers as the reciprocal of the highest serum dilution, the OD value of which was 2.1-fold higher than that of the negative control.

### RVFV neutralization assay

The neutralizing activity of the mice serum was measured through a microneutralization assay as previously described with few modifications (Wichgers Schreur et al., 2017; Bouhaddou et al., 2020). Serum samples were heated at 56°C for 30 min before use. The 3-fold serial dilutions of sera were incubated with 100 TCID_50_ of rMP-12-eGFP in 96-well plates at 37°C for 1 h. After that, 20,000 Vero E6 cells were added to each well and incubated at 37°C with 5% CO_2_ for 48 h. Then, the supernatant was discarded, and the cells were fixed with 4% paraformaldehyde for 3 h. Afterward, the cells were washed with PBS three times and immune-stained with a DAPI counterstain (Thermo Scientific, Waltham, MA, United States). The counts of infected cells (eGFP) and total cells (DAPI) were detected using the Celigo (Nexcelom, Boston, MA, United States) imaging cytometer. We defined the focus reduction neutralization test (FRNT_50_) as the reciprocal of serum dilution, which inhibits 50% of viral infection compared with virus-only wells.

### ELISpot

Rift valley fever virus-specific cellular immune responses in mice were evaluated through mouse IFN-γ and IL-2 ELISpot Kits (MabTech, Stockholm, Sweden). In brief, 1 × 10^5^ splenocytes of vaccinated mice were stimulated with 14 peptides (2 μg/ml), which were identified from RVFV Gn and Gc proteins previously (Warimwe et al., 2013). Subsequently, the cells seeded in precoated ELISpot plates at 37°C in a 5% CO_2_ incubator for 16 h. After the plates were rinsed five times with PBS, biotinylated anti-mouse IFN-γ and IL-2 were added to the plates and incubated at room temperature for 1 h. After five rinses, the plates were rinsed incubated with streptavidin-HRP at room temperature for 1 h. After the plates rewashed, TMB substrate was applied to develop spots for ELISpot. Eventually, the plates were rinsed sufficiently with water to stop color development and allowed to dry without light. The spots were recorded utilizing an ELISPOT reader (AID GmbH, Strassberg, Germany).

### Intracellular cytokine staining

BALB/c mouse splenocytes were prepared by the method of grinding. The erythrocytes were washed several times after lysis, and 2 × 10^6^ splenocytes were seeded into 24-well plates and blocked cytokine secretion by BD GolgiStopTM. Meanwhile, the splenocytes are stimulated by 2 μg/mL of the 14 peptides described above at 37°C. After stimulation for 6 h, the splenocytes were washed with PBS and stained with the LIVE/DEAD™ Fixable Near-IR Dead Cell Stain Kit (Invitrogen, Waltham, MA, United States). Then, the cells were washed again, blocked with anti-CD16/32 (Biolegend, San Diego, CA, United States), and stained with a mixture of antibodies, including anti-mouse CD3 (PerCP/Cyanine5.5, Biolegend, San Diego, CA, United States), anti-mouse CD19 (APC/Cyanine, Biolegend, San Diego, CA, United States), anti-mouse CD4 (Alexa Fluor 700, Biolegend, San Diego, CA, United States), anti-mouse CD8a (Brilliant Violet 510™, Biolegend, San Diego, CA, United States), and anti-mouse CD107a (Brilliant Violet 421™, Biolegend, San Diego, CA, United States) diluted in Cell Staining Buffer (Biolegend). After simultaneous fixation and permeabilization with Cytofix/CytopermTM Fixation and Permeabilization Solution (BD), the cells were washed with Perm/Wash buffer (BD Biosciences, Franklin Lakes, NJ, United States) and PBS and stained with anti-mouse TNF-α (APC, Biolegend, San Diego, CA, United States), anti-mouse IFN-γ (PE, Biolegend, San Diego, CA, United States), anti-mouse IL-4 (Brilliant Violet 605™, Biolegend, San Diego, CA, United States), and anti-mouse IL-2 (PE/Cyanine7, Biolegend, San Diego, CA, United States). Finally, the cells were washed and resuspended with PBS. The data were collected on a FACS Canto™ flow cytometer (BD Biosciences, Franklin Lakes, NJ, United States).

The draining inguinal lymph nodes (LNs) from BALB/c mice were prepared by the same method with splenocytes, and 2 × 10^6^ cells were stained using the LIVE/DEADTM Fixable Near-IR Dead Cell Stain Kit (Invitrogen, Waltham, MA, United States) and blocked with anti-mouse CD16/32. Subsequently, the cells were stained with CD19 (Brilliant Violet 605™, Biolegend, San Diego, CA, United States), anti-mouse CD4 (FITC, Biolegend, San Diego, CA, United States), anti-mouse CD3 (PerCP/Cyanine5.5, Biolegend, San Diego, CA, United States), anti-mouse Fas (APC, Biolegend, San Diego, CA, United States), anti-mouse GL7 (PE, Biolegend, San Diego, CA, United States), anti-mouse PD-1 (Brilliant Violet 421™, Biolegend, San Diego, CA, United States), and anti-mouse CXCR5 (PE/Cyanine7, Biolegend, San Diego, CA, United States). After staining, the cells were fixed, resuspended with PBS, and collected using a BD FACS Canto™ flow cytometer.

### Quantitative RT-PCR

We determined viral loads in the spleens and livers of A129 mice by q PCR. A total of 30 mg of samples were homogenized, and RNA was extracted with an RNeasy Mini Kit (Qiagen, Dusseldorf, North Rhine-Westphalia, Germany). The total RNA amount was measured using spectrophotometer, and all RNA samples uniformly were diluted to 400 ng/μL prior to reverse transcription. Reverse transcription was performed using PrimeScript™ RT Master Mix (Takara, Dalian, Liaoning, China). qPCR was conducted using TaqMan™ Universal Master Mix II (Thermo Scientific, Waltham, MA, United States) with the following primers and probes as previously described: forward primer 5′-GAAAATTCCTGAAACACATGG-3′, reverse primer 5′-ACTTCCTTGCATCATCTGATG-3′, and probe FAM-CAATGTAAGGGGCCTGTGTGGACTTGTG-BHQ1-3′(Bird et al., 2007). The standard curve was generated from a plasmid containing partial cDNA of the RVFV L gene and used to normalize the viral RNA amount.

### Histopathology and immunohistochemistry

The spleen and liver tissues of A129 mice were collected and fixed in 4% paraformaldehyde solution at RT for 72 h, embedded in paraffin, and sectioned. Hematoxylin and eosin (H&E) stain was used to identify microscopic lesions. For immunohistochemistry, RVFV Gn protein as detected using a RVFV Gn protein specific monoclonal antibody. Images were captured using a Panoramic 250 FLASH and were processed using software Slide Viewer.

### Statistical analysis

All statistical analyses in this study were performed through software Graphpad Prism 8.0, and data are displayed as the mean ± SEM. One-way ANOVA with Tukey’s multiple comparison test and two-way ANOVA with Sidark’s multiple comparisons test were carried out for multiple group (>2) comparisons, and a two-tailed *t*-test was performed for comparison of two groups. The Kaplan–Meier survival curves were analyzed using the Log-rank (Mantel-Cox) test. *p* < 0.05 was considered to indicate a significant difference. ^*^p < 0.05, ^**^p < 0.01, ^***^p < 0.001, and ^****^p < 0.0001, ns, no significance.

## Results

### Construction and characterization of Ad5-GnGcopt

**Figure 1:**
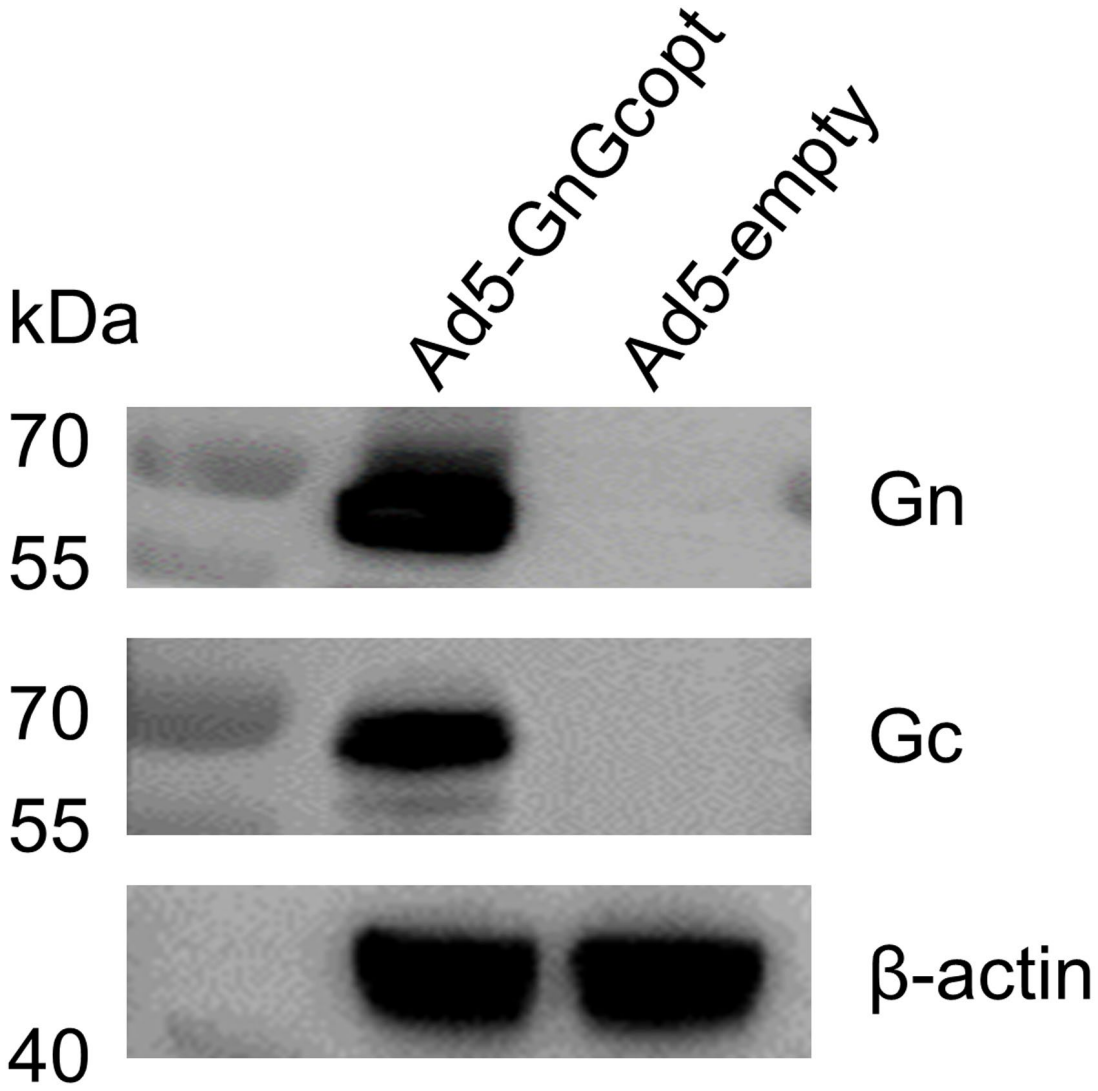
Characterization of rift valley fever virus (RVFV) vaccine Ad5-GnGcopt. Vero E6 cells were infected with Ad5-GnGcopt and Ad5-empty at a multiplicity of infection (MOI) of 10. The cells were collected 48 h after infection, and Western blotting was performed to identify the expression of antigenic proteins Gn and Gc.

The RVFV MP-12 strain glycoproteins Gn and Gc (accession number DQ380208.1) were inserted into E1/E3 deleted replication-defective Ad5 vector-based platform. The Gn and Gc genes were codon-optimized to enhance antigen expression in mammalian cells and the tPA signal peptide was employed to substitute the original viral signal peptide. Ad5-GnGcopt was successfully rescued and propagated in HEK 293 cells. The expression of Gn and Gc proteins in Vero E6 cells infected with Ad5-GnGcopt at an MOI of 10 was identified by Western blot (Figure 1).

### Ad5-GnGcopt induces strong and durable humoral immune responses in mice

**Figure 2:**
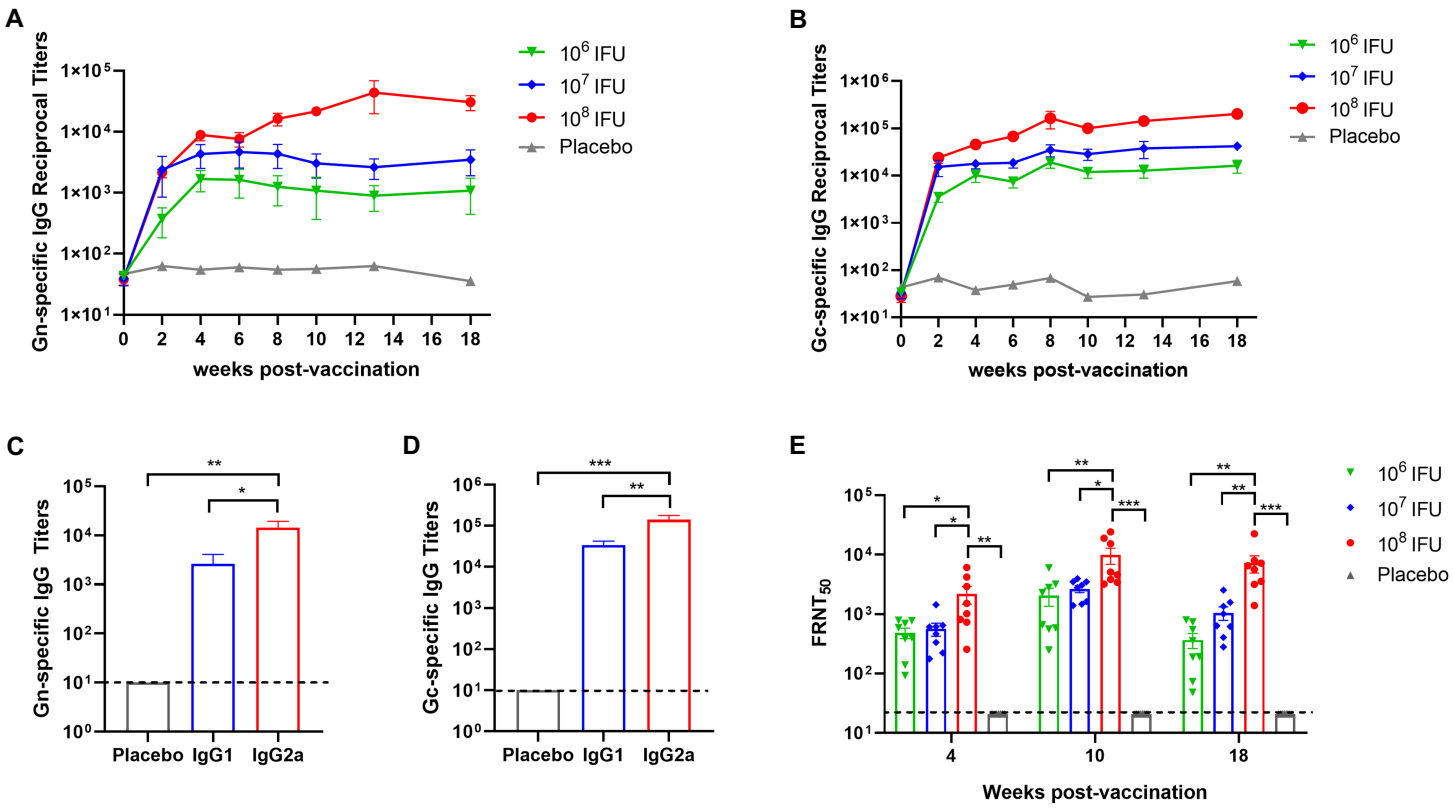
Durable humoral immune response elicited by Ad5-GnGcopt in BALB/c mice. BALB/c mice (*n* = 8) were intramuscularly immunized with a single dose of Ad5-GnGcopt (10^6^, 10^7^, or 10^8^ IFU) or placebo. Sera were collected at 2, 4, 6, 8, 10, 13, and 18 weeks after vaccination. RVFV **(A)** Gn- and **(B)** Gc-specific IgG antibody titer was determined by ELISA. **(C,D)** Sera from the high-dose group collected on day 28 after immunization determined **(C)** Gn- and **(D)** Gc-specific IgG subclasses. **(E)** Serum samples collected 4, 10, and 18 weeks after vaccination were subjected to neutralizing antibody titers detection. Data are indicated as mean ± SEM. One-way ANOVA with multiple comparison tests calculated *p* values. ^*^p < 0.05, ^**^p < 0.01, ^***^p < 0.001.

To evaluate the immune response induced by Ad5-GnGcopt, 4–6 weeks old female BALB/c mice (*n* = 8) received a single dose of either 10^6^ IFU (low dose), 10^7^ IFU (middle dose), or 10^8^ IFU (high dose) of Ad5-GnGcopt or placebo *via* the intramuscular route at week 0. Serum samples were harvested 2, 4, 6, 8, 10, 13, and 18 weeks after vaccination for detection of Gn- and Gc-specific IgG, IgG1, IgG2a, and RVFV-specific neutralizing antibody. The ELISA results show that Ad5-GnGcopt induces viral-specific IgG response, which is dose dependent. And even a low dose produces IgG responses efficiently. The titer of Gc-specific IgG in serum was generally higher than that of Gn at different time points in each group. More importantly, the binding antibodies were maintained at high levels up to 18 weeks after immunization, implying that Ad5-GnGcopt could induce long-lasting immune responses (Figures 2A,B). Additionally, Gn- and Gc-specific IgG1 and IgG2a were also detected at 4 weeks after immunization in the high-dose group. Furthermore, our findings show that the vaccine could induce not only IgG1 but also IgG2a responses and that the titer of IgG2a was significantly higher than that of IgG1, implying a Th1-biased immune response (Figures 2C,D). Neutralizing antibody (NAb) titers were detected using a 50% focus reduction neutralization test. In line with expectations, Ad5-GnGcopt also induced the NAb in a dose-dependent manner, and a low dose of Ad5-GnGcopt can induce good and long-lasting neutralizing antibody responses (Figure 2E).

### Ad5-GnGcopt induces strong cellular immune responses in BALB/c mice

**Figure 3:**
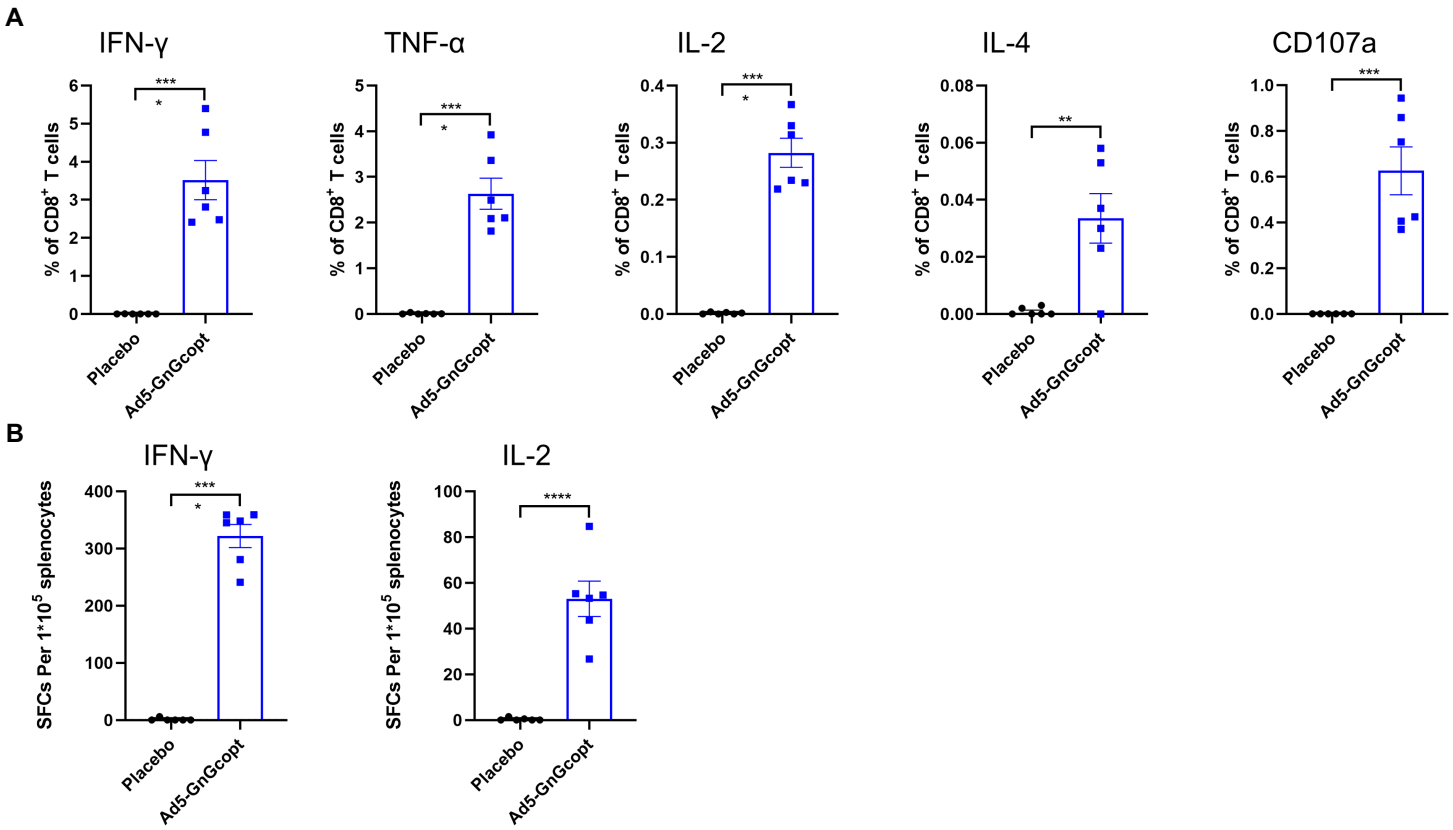
The cellular immune responses elicited by Ad5-GnGcopt in BALB/c mice. Four to six-week-old BALB/c mice (*n* = 6) were immunized with a single injection of Ad5-GnGcopt (10^8^ IFU) or placebo. The splenocytes were collected 2 weeks after immunization. **(A)** The percentages of CD8^+^T cells secreting IFN-γ, TNF-α, IL-2, IL-4, and CD107a determined using intracellular cytokine staining assay. **(B)** IFN-γ and IL-2 secretion from mouse splenocytes detected by ELIspot. Data are shown as means ± SEM. Comparisons based on Student’s unpaired *t*-test with ^**^p < 0.01, ^***^p < 0.001, and ^****^p < 0.0001.

For further characterizing the cellular immune response induced by Ad5-GnGcopt, intracellular cytokine staining assays and ELISpot were performed. Splenocytes of BALB/c mice from high-dose group were harvested at 2 weeks post-vaccination and evaluated for their capacity for secreting cytokines after restimulating with RVFV Gn and Gc peptides *in vitro*. Our results demonstrate that CD8+ T cells secreting IFN-γ, TNF-α, IL-2, and IL-4 were significantly more abundant in Ad5-GnGcopt-immunized mice than in placebo-immunized mice. The CD8+ T cells were also significantly induced to secrete CD107a, which is a marker of degranulation and cytotoxic activity of cytotoxic CD8+ T cell (Wang et al., 2016; Figures 3A,B), suggests that Ad5-GnGcopt can induce cellular immune responses in BALB/c mice efficiently.

### Ad5-GnGcopt triggers robust GC responses in lymph nodes

**Figure 4:**
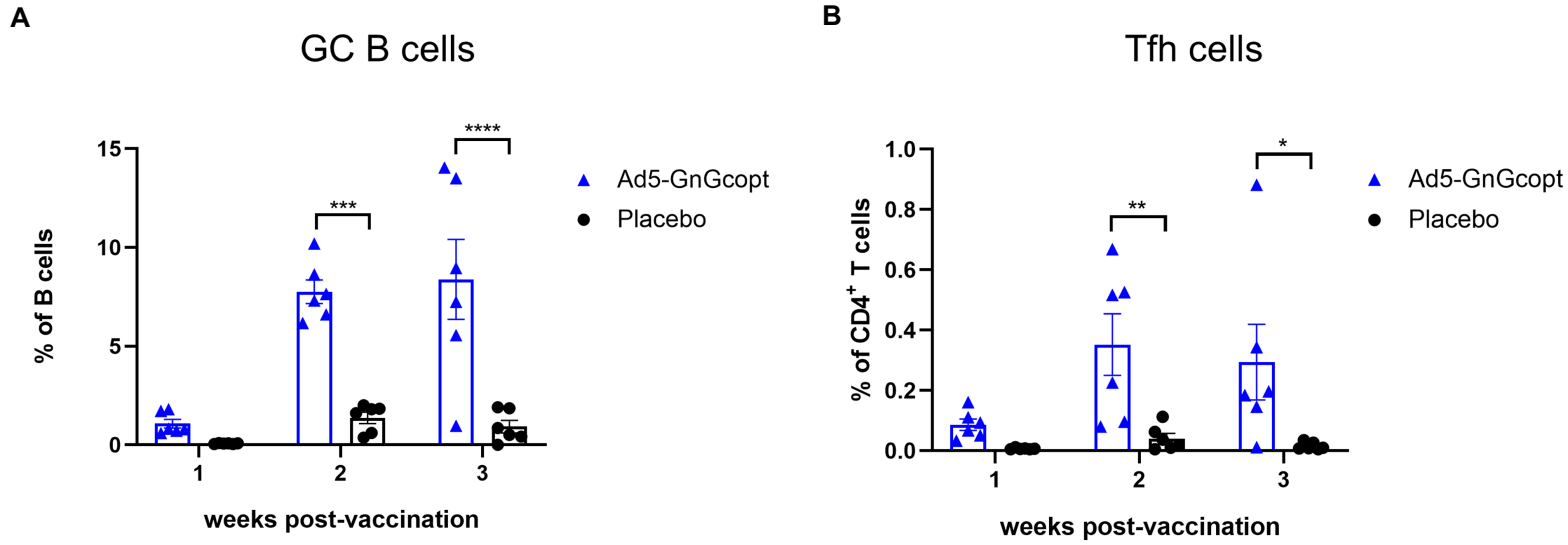
Ad5-GnGcopt efficiently stimulates strong GC B cells and Tfh cells responses. BALB/c mice (*n* = 18) were immunized with a single dose of Ad5-GnGcopt (10^8^ IFU) or placebo *via* the intramuscular route. Six mice from each group were euthanized, and the inguinal lymph nodes were collected at 1, 2, and 3 weeks after immunization. **(A)** The proportion of GC B cells (CD3-CD19^+^GL7^+^Fas^+^) and **(B)** the frequency of Tfh cells defined as live, CD3^+^CD4^+^PD-1^+^CXCR5^+^ in each group as determined using flow cytometry. Data are displayed as means ± SEM. Comparisons based on two-way ANOVA with Sidark’s multiple comparisons test with ^*^p < 0.05, ^**^p < 0.01, ^***^p < 0.001, and ^****^p < 0.0001.

The germinal center (GCs) is an important microanatomical site for B cell mutation and antibody affinity maturation. During the immune response, GCs are tightly regulated by follicular helper T cells (Tfh), which enable B cell survival, proliferation, and differentiation through the delivery of costimulatory molecules and cytokines (Allen et al., 2007; Crotty, 2019). We inferred the formation of superior GC in secondary lymphoid organs facilitates Ad5-GnGcopt to induce the robust immune responses. To test our hypothesis, BALB/c mice were vaccinated intramuscularly with Ad5-GnGcopt (10^8^ IFU) in a single dose way. Inguinal LNs were harvested 1, 2, and 3 weeks after vaccination and subjected to GC B and Tfh cell detection using flow cytometric analysis. The results indicated that the percentages of total GC B cells in Ad5-GnGcopt vaccinated mice were all considerably higher than those of placebo-vaccinated mice, and the response could reach a high level at 2 weeks post-immunization and persist for at least 3 weeks (Figure 4A). Similarly, a higher proportion of Tfh cells was also identified in inguinal LNs (Figure 4B). These results suggest that Ad5-GnGcopt induces relatively good GC and Tfh reactions lasting at least 3 weeks after immunization.

### Ad5-GnGcopt vaccination induces protection against RVFV challenge in A129 mice

**Figure 5:**
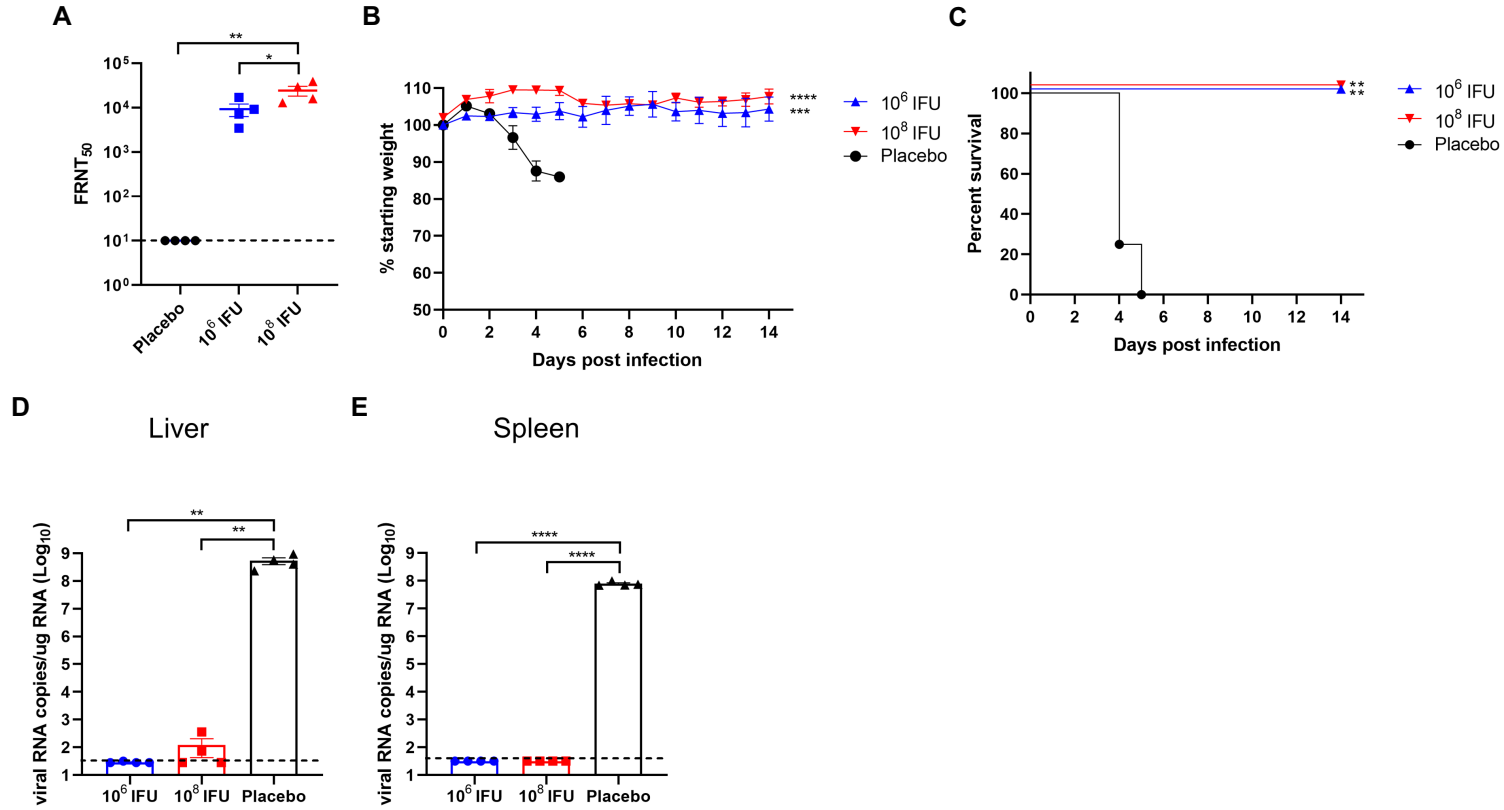
Protection efficacy of Ad5-GnGcopt against RVFV challenge in IFN receptor-deficient mice. A129 mice (*n* = 4) were injected with one dose of 10^6^ and 10^8^ IFU of Ad5-GnGcopt or placebo *via* the intramuscular route. Four weeks after vaccination, mice were challenged intraperitoneally with 2 × 10^4^ TCID_50_ of the RVFV rMP-12 strain. **(A)** Sera collected at 4 weeks after vaccine immunization (before virus challenge) were examined for neutralizing antibodies using infectious RVFV. **(B)** Body weight change in mice after RVFV challenge. **(C)** The mortality and survival curves of mice. **(D,E)** Viral loads in livers **(D)** and spleens **(E)** of challenged mice were determined by qRT-PCR at the time of death (placebo group) or 14 days after the challenge (vaccine immunized groups). Data are displayed as means ± SEM. Comparisons for FRNT_50_ and viral RNA copies based on two-way ANOVA with Sidark’s multiple comparisons test with ^*^p < 0.05, ^**^p < 0.01, ^***^p < 0.001, and ^****^p < 0.0001. The weight curves were analyzed using one-way ANOVA (Multiple comparisons) test with ^*^p < 0.05, ^**^p < 0.01, ^***^p < 0.001, and ^****^p < 0.0001. The Kaplan–Meier survival curves were analyzed using the Log-rank (Mantel-Cox) test with ^*^p < 0.05, ^**^p < 0.01, ^***^p < 0.001, and ^****^p < 0.0001.

**Figure 6:**
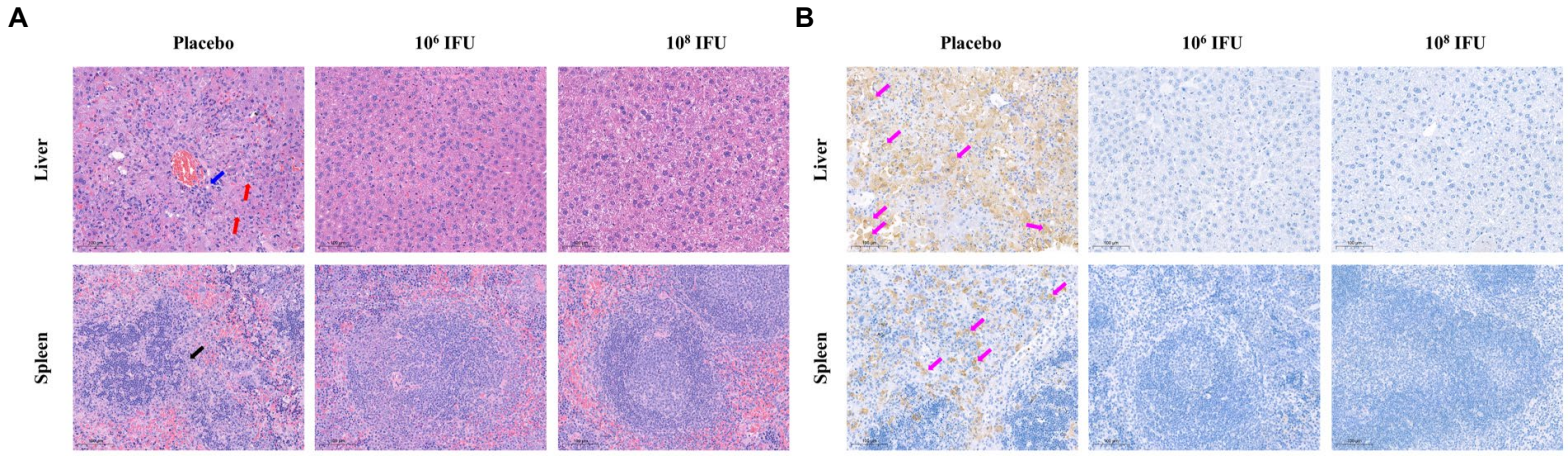
Representative histopathology and immunohistochemistry of livers and spleens in RVFV-infected IFN receptor-deficient mice. The livers and spleens from the placebo group (at the time of death) and vaccine-immunized groups (14 days after challenge) were collected and analyzed with histopathological and immunohistochemical assays. **(A)** Representative histopathology (H&E) of livers and spleens in RVFV-infected mice. Hepatocyte steatosis is indicated by red arrows, hepatocyte necrosis is indicated by blue arrows, and splenic nodules atrophy is indicated by black arrows. Scale bar, 100 μm. **(B)** Representative immunohistochemistry (IHC) of liver and spleen tissues with RVFV Gn-specific monoclonal antibodies. Purple arrows indicate RVFV-positive signals. Scale bar, 100 μm.

A129 mouse is a common animal model for studying virology, pathology, and immunology and for screening antiviral vaccines and medicines of many viruses, such as infection with Zika virus or RVFV (Ikegami et al., 2006; Zhu et al., 2017; Makris and Pavord, 2022). Therefore, A129 mouse was selected to explore the *in vivo* protection efficacy of Ad5-GnGcopt against RVFV challenge. In this experiment, A129 mice inoculated a single dose of 10^6^ IFU, 10^8^ IFU Ad5-GnGcopt, or placebo *via* intramuscular route, and the pre-challenge sera (4 weeks after vaccination) were collected for NAb detection. Our results show that both the10^6^ IFU and 10^8^ IFU groups produced high NAb titers against RVFV in A129 mice as determined by live virus neutralization assays (Figure 5A). All mice were challenged intraperitoneally with 2 × 10^4^ TCID_50_ of RVFV rMP12 strain 4 weeks after vaccination. The body weight of each mouse was recorded daily for 14 days after RVFV challenge. The results indicated that the body weights increased steadily for both the10^6^ IFU and 10^8^ IFU groups decreased significantly for the placebo group (Figure 5B). All animals in the placebo group died within 4–5 days after the challenge, whereas mice in the vaccine-immunized group remained healthy and survived at the end of the experiment (Figure 5C). At 14 days after challenge, all surviving mice in the Ad5-GnGcopt immunized groups were euthanized, and the livers and spleens were collected for viral RNA loads, histopathological, and immunohistochemical assays. The assay about viral RNA loads indicated that the placebo-inoculated mice became infected, with 10^8.73^ and 10^7.9^ viral RNA copy equivalents per microgram of total RNA in livers and spleens at the time of death, respectively. By contrast, Ad5-GnGcopt-immunized mice were protected from RVFV infection, with almost no viral RNAs detected at the time of euthanasia (14 days after the challenge; Figures 5D,E). Histopathological examination indicated that severe microscopic liver lesions were observed in placebo mice at the time of death, characterized by hepatocyte steatosis, inflammatory cell infiltration, and scattered necrotic foci. Meanwhile, scattered splenic nodules atrophy was observed in splenic tissues. However, there were no pathological changes observed in the liver and spleen samples from all Ad5-GnGcopt vaccinated animals at the end of experiment (Figure 6A). Similarly, immunohistochemical assays detected many RVFV-positive signals in placebo mice, whereas no RVFV were detected in the livers and spleens fromAd5-GnGcopt vaccinated mice (Figure 6B). The above results demonstrate that a single low-dose immunization of Ad5-GnGcopt prevents RVFV replication in the liver and spleen and efficiently protects mice from lethal infection.

## Discussion

Rift valley fever virus is an important pathogen that significantly affects humans and animals, causing huge economic losses. Vaccination is one of the most economical and effective means to control the occurrence and spread of diseases. And the ideal vaccine plays an antiviral role through inducing humoral and cellular immune responses rapidly. The RVFV glycoproteins Gn and Gc, mediating viral entry and containing the crucial epitopes for neutralizing antibodies (Besselaar and Blackburn, 1992), are considered the primary antigen for developing RVFV vaccines (Faburay et al., 2017). On the basis of these results, numerous types of RVFV vaccine have emerged, including inactivated vaccines, live-attenuated vaccines, recombinant proteins vaccines, DNA vaccines (Chrun et al., 2018), recombinant viral vectors such as recombinant adenovirus vectors (Holman et al., 2009; Stedman et al., 2019; Bian et al., 2022), and recombinant modified vaccinia virus Ankara (MVA; Lopez-Gil et al., 2013; Calvo-Pinilla et al., 2020), among others. Although these vaccines show different degrees of immunogenicity and protective efficacy in different animal models, shortcomings or limitations were also observed.

The pre-existing immunity to the HAdV-5 vector was a big obstacle to the practical application of Ad5-GnGcopt, since high preexisting immunity was shown to diminish the humoral and cellular immune response induced by the intramuscular HAdV-5 vector vaccine (Zhu et al., 2015, 2020). Whereas, there are several studies indicating that the pre-existing immunity to the HAdV-5 vector may not influence the mucosal immunization HAdV-5 vector vaccine (Croyle et al., 2008; Choi et al., 2012; Liebowitz et al., 2020). These data offer several novel immunization ways to overcome the pre-existing immunity to the HAdV-5 vector, such as intranasal administration, pulmonary atomization, and eye drops. Especially for RVFV, one of the clinical signs of RVFV infectious is ocular disease (0.5–2% of patients). The eye drop delivery of Ad5-GnGcopt may prevent ocular disease in patients infected with RVFV.

In this research, we constructed a replication-incompetent recombinant adenovirus RVF vaccine Ad5-GnGcopt and demonstrated that this vaccine could elicit antibody and cellular immune responses in BALB/c mice effectively (Figures 2–4). Moreover, a single low dose of Ad5-GnGcopt (10^6^ IFU) can protect A129 mice against lethal RVFV challenge. Since RVF vaccinations are often administered during outbreaks, it is significantly more difficult to receive two shots than one shot. Hence, our findings suggest that Ad5-GnGcopt might be used as a potential vaccine candidate in human clinical trials.

Previous studies have elucidated that interferon-α/β receptor-deficient mice are susceptible to attenuated RVFV strains MP-12 (Bouloy et al., 2001). This animal model has been widely used for evaluation of RVF vaccines (Ikegami et al., 2006; Bouhaddou et al., 2020; Makris and Pavord, 2022). In the present research, we also used this animal model to assess the protective efficacy of the vaccine candidate Ad5-GnGcopt. Our studies *in vivo* demonstrate that Ad5-GnGcopt could induce high titers of neutralizing antibodies in this mouse model, and even a low dose of 10^6^ IFU vaccination could inhibit virus replication in the tissues and protect mice from a lethal RVFV challenge. During the experiment, mice injected with a placebo displayed obvious clinical symptoms, with 13.8–16.6% of body weight lost and dying within 4–5 days after the challenge. Viral loads in their livers and spleens reached 10^8.73^ and 10^7.9^ per microgram of total RNA at the time of death, respectively. These findings indicate that mice in the placebo group died of RVFV infection. Obvious pathological changes were observed in their livers and spleens, and the viral Gn protein was also detected in respect to those lesions. By contrast, the Ad5-GnGcopt immunized mice kept healthy without clinical sign, showed a steady increase in body weight, and survived until the end of the experiment. Moreover, almost no viral genome could be detected in livers and spleens at the time of euthanasia, indicating that RVFV was cleared. In accordance with this result, no obvious microscopic lesions could be observed in the livers and spleens of the immunized mice (Figures 5, 6).

Our studies demonstrate that Ad5-GnGcopt can induce good immune responses in BABL/c and A129 mice. Since many animals are susceptible to RVFV and Ad5-based vaccines have been developed in ruminants against bluetongue virus or Peste des Petits Ruminants Virus (Rojas et al., 2014; Martin et al., 2015), Ad5-GnGcopt immunogenicity and efficiency requires further validation in other animal models such as in ruminants. Another limitation of our study is that the attenuated RVFV strain MP-12 was used to evaluate the protective activity of Ad5-GnGcopt *in vivo*. And the RVFV virulent strains employed in the further challenge experiments will improve the comprehensiveness of the experimental data and potentially provide support toward their validation. What is more, A129 mice model challenged by RVFV strain MP-12 is also a main limitation in our study. Since innate immune responses are impaired in A129 mice, they might influence subsequent adaptive immune responses. This would not objectively reflect the true protective effect of Ad5-GnGcopt. And whether the symptoms of these mice after RVFV infection are the same as those of humans or ruminants still need to be verified.

In conclusion, the Ad5-based replication incompetent RVF vaccine exhibited good immunogenicity and protective activity in BABL/c and A129 mice, suggesting that Ad5-GnGcopt might be a potential candidate vaccine against RVFV.

## Data availability statement

The datasets presented in this study can be found in online repositories. The names of the repository/repositories and accession number(s) can be found in the article/Supplementary material.

## Ethics statement

The animal study was reviewed and approved by the Animal Welfare and Ethics Committee of the Academy of Military Medical Sciences, China.

## Author contributions

WC, JL, and CY designed the experiments. MH, TB, GF, YC, TF, CZ, and SL performed the experiments. MH and TB analyzed the data. MH wrote the manuscript. All authors contributed to the article and approved the submitted version.

## Funding

This research was supported by the ZJU-Hangzhou Global Scientific and Technological Innovation Center (02120000-K020130001).

## Conflict of interest

The authors declare that the research was conducted in the absence of any commercial or financial relationships that could be construed as a potential conflict of interest.

## Publisher’s note

All claims expressed in this article are solely those of the authors and do not necessarily represent those of their affiliated organizations, or those of the publisher, the editors and the reviewers. Any product that may be evaluated in this article, or claim that may be made by its manufacturer, is not guaranteed or endorsed by the publisher.
